# Elite Players Invest Additional Time for Making Better Embodied Choices

**DOI:** 10.3389/fpsyg.2022.873474

**Published:** 2022-06-06

**Authors:** Matthias Hinz, Nico Lehmann, Lisa Musculus

**Affiliations:** ^1^Department of Sport Science, Institute III, Otto von Guericke University, Magdeburg, Germany; ^2^Department of Neurology, Max Planck Institute for Human Cognitive and Brain Sciences, Leipzig, Germany; ^3^Department of Performance Psychology, Institute of Psychology, German Sport University, Cologne, Germany

**Keywords:** decision making, decision time, decision confidence, team-handball, expertise

## Abstract

Expert athletes are determined to make faster and better decisions, as revealed in several simple heuristic studies using verbal reports or micro-movement responses. However, heuristic decision-making experiments that require motor responses, also being considered as the embodied-choice experiments, are still underrepresented. Furthermore, it is less understood how decision time and confidence depend on the type of embodied choices players make. To scrutinize the decision-making processes (i.e., decision time, decision confidence), this study investigated the embodied choices of male athletes with different expertise in a close-to-real-life environment; 22 elite (*M*_age_ = 17.59 yrs., *SD* = 3.67), and 22 amateur (*M*_age_ = 20.71 yrs., *SD* = 8.54) team handball players performed a sport-specific embodied-choice test. Attack sequences (*n* = 32) were shown to the players, who had to choose between four provided options by giving a respective sport-specific motor response. We analyzed the frequencies of *specific choices* and the *best choice*, as well as the respective decision time and decision confidence. Elite and amateur players differed in the frequencies of *specific* choices (i.e., forward/tackling; passive blocking), and elite players made the *best choice* more often. Slower decision times of elite players were revealed in *specific choices* and in *best choices*, the confidence of decisions was rated equally high by both player groups. Indications are provided that elite players make better choices rather slower, instead of faster. We suppose this is due to specific sensorimotor interactions and speed-accuracy-tradeoffs in favor of accuracy in elite players. Our findings extend expert decision-making research by using an embodied-choice paradigm, highlighting considerations of decision time and confidence in future experiments.

## Introduction

Extensive research in cognitive psychology in sports attributes superiority in decision-making to expert athletes (Mann et al., 2007; Travassos et al., 2013; Araújo et al., 2019; Silva et al., 2020). In team sports, characterized by the highly dynamic, frequently changing, and time-pressured situations (Raab, 2012; Belling et al., 2015; Musculus et al., 2018), the decision accuracy, time, and confidence are crucial indicators of *how* decisions are made as they are. In particular, these highly complex situations challenge an athlete to make fast and accurate decisions and to confidently rely on their choices even if alternative options evolve quickly through the dynamic changes.

Real-world decision-making is embedded in a complex and uncertain context and, especially in team sports, the information processing of athletes underlies time and information constraints (Travassos et al., 2012; Kinrade et al., 2015). Therefore, it appears necessary to consider a domain-specific environment in decision-making experiments, where sensorimotor interactions in athletes' decision-making processes can unfold (Burk et al., 2014; Raab, 2014; Kalén et al., 2021). By doing so, studies in netball (Bruce et al., 2012) and soccer (van Maarseveen et al., 2018a) compared the decision-making performances from uncoupled perception-action tests (using verbal reports, button press, or micro-movements as responses) with those from actual on-field performances tests. The obtained results showed no correlation between the performance outcomes in both conditions which suggests that uncoupled response methods in perceptual-cognitive experiments might not be sufficient enough to predict actual in-game decision-making (Travassos et al., 2013). However, there are other voices in perceptual-cognitive research determining uncoupled perception-action responses as either similar (Farrow et al., 2005), or even more accurate (Ranganathan and Carlton, 2007) than coupled perception-action responses for the targeted research goal. For example, when an observer remains static during an experiment (e.g., in a computer-based setting), the perceptual information pick up is still linked to the motor regions of the brain (Aglioti et al., 2008). Another exemplary experiment in team handball (Huesmann et al., 2021) compared goalkeepers' anticipation performances in an artificial (verbal responses) and a simulated (motor responses) condition. The authors found higher prediction accuracies of the goalkeepers in the artificial, verbal response condition. The findings in these studies suggest that the evidence on whether considering motor components in decision-making experiments is beneficial for capturing expertise effects seems mixed, therefore, requiring further clarification.

### Embodied Choices in Sports

To further facilitate our understanding of expert decision-making behavior, the framework of simple heuristics (Raab, 2012) containing rules of thumb for the explanation of decisions has been expanded from an embodied cognition perspective. This offers possibilities to account for the interaction of cognitive and motor processes throughout decision-making processes and for the motor execution of choices in particular (Raab, 2017). Therefore, the integrative concept of embodied choices (Lepora and Pezzulo, 2015) aims for the explanation of complex sports behavior, “when limited time and resources force athletes to decide quickly between two or more options” (Raab, 2017, p. 35). The application of embodied choices enables to incorporate key mechanisms in decision-making processes, such as action preparation and commitment (Lepora and Pezzulo, 2015). Action preparation strategies are stated to alleviate delays in enacting a choice and also to amend the termination of a decision. Commitment effects are created when the dynamics of an action changes the prospects, therefore, affecting the initially preferred choice. For this reason, embodied-choice paradigms seem to suit better to depict fast and accurate responses in situations that are ecologically valid (Lepora and Pezzulo, 2015).

To evaluate the mechanisms behind embodied choices, it seems worthwhile to assess not only the accuracy of decisions but also the time of the decisions made being determined to discriminate between expertise levels (Travassos et al., 2013). Additionally, the confidence of the decisions is another fundamental aspect of decision-making, especially for multiple-choice alternatives (Ratcliff and Starns, 2013).

To our best knowledge, only one study within the embodied-choice framework exists that examined embodied choices concerning choice quality. van Maarseveen et al. (2018b) used a 3 vs. 3 pick-and-roll basketball play with three types of defensive play, and the participants assumed the role of the ball carrier. Analyses of the tactical decisions made, and the quality of decisions (determined by two coaches as correct or incorrect decisions), showed that the players made different decisions on either the left or right side of the court, and when they faced different defensive plays. Another embodied examination in team handball compared expert and near-expert players' decision-making performances in a cave automatic virtual environment under real-world conditions (Magnaguagno and Hossner, 2020). Attack sequences were presented showing 1 vs. 1 situations of a defending teammate and a backcourt attacker, and players were required to give an embodied defense response. The authors found expertise effects in response correctness, showing that expert players responded more appropriately on a lost 1 vs. 1 duel of the respective defending teammate than the near-expert players. To the best of our knowledge, only one sports study assessed decision confidence in an embodied-choice setting testing with sport-specific motor responses (Hinz et al., 2021). Thus, further research is needed to better understand how decision time and confidence are affected, not only by the choices made and their quality, but also by differing levels of expertise.

### Time and Confidence in Sports Decision-Making

In addition to that, decision-making theories often model response time and the confidence of a decision because they are regarded as crucial measures of decision performance (Ratcliff et al., 2016). There are several studies that investigated decision time in complex team sports settings so far. To mention a few, the *in situ* study of van Maarseveen et al. (2018b) in basketball also measured the decision time of the correct and incorrect decisions within the 3 vs. 3 pick-and-roll plays. The average execution time of correct and incorrect decisions differed with almost obtained significance, implying that better decisions seem to be made in a faster way. On the contrary, a heuristic study in team handball using verbal responses, i.e., a non-motor response, determined decision time as a performance-discriminating factor between experts, near-experts, and non-experts (Raab and Laborde, 2011). In soccer, the studies of Vaeyens et al. (2007a,b) assessed decision time by the execution of movement-based responses on varying offensive patterns of play. The results also demonstrated that successful players achieved faster decision times within the presented decision scenarios. From a cognitive perspective, a decision can be made faster by sacrificing accuracy, and a decision can also be more accurate with sacrificed decision speed (speed-accuracy tradeoff) (Ratcliff et al., 2016). The evidence from the bespoken studies indicates that decision time seems to play an important role in the underlying decision-making processes. To date, the number of decision-making studies assessing decision time is rather manageable (Travassos et al., 2013), especially when considering that many examinations used varying response and stimulus presentation methods. Still, more clarification is required about the correlation between decision-making performance and decision time in complex, near-game experiments using representative task designs.

To be able to further estimate not only the speed and the accuracy of a decision, it also appears worthwhile to assess the confidence of the judgment of a decision (Ratcliff et al., 2016; Seale-Carlisle et al., 2019). Only a few studies in the team sports domain exist that have put the focus on the athletes' confidence in decisions. In a basketball study by Hepler and Feltz (2012), they applied a video-based cognitive decision-making test. The results for decision confidence (defined as the subjective estimation of the success of a decision made) indicate that basketball players were more confident in better options and choices. A study with young expert players in soccer found correlations between decision confidence and motor confidence (both on the Likert-type scale), which was defined as the subjective estimation of the ability to execute a respective option (Musculus et al., 2018).

Together, embodied-choice paradigms are needed to not only facilitate our understanding of real-world behavior of athletes but also to validly investigate the mechanisms underlying embodied choices. Decision time and confidence in complex sports behavior are relevant parameters that have been strongly recommended by cognitive scientists to be taken into account in decision-making experiments (Ratcliff and Starns, 2013; Ratcliff et al., 2016). This might hold for the team sports in particular, given the time constraints and uncertainty through dynamic changes throughout a game. Despite the highlighted advantages of embodied cognition paradigms, there are only a few studies which investigate the decision time and confidence for embodied choices. Thus, we examined what kind of embodied choices elite and amateur team handball players made, and how decision time and decision confidence are affected by the respective choices of players.

### This Study

To summarize, this study extends current theorizing and the empirical state-of-the-art by testing embodied choices in a near-game environment in team handball, which allows us to analyze (1) what choices are made through capturing a sport-specific motor response, and (2) how decision time and confidence differ based on the choices made.

**Figure 1:**
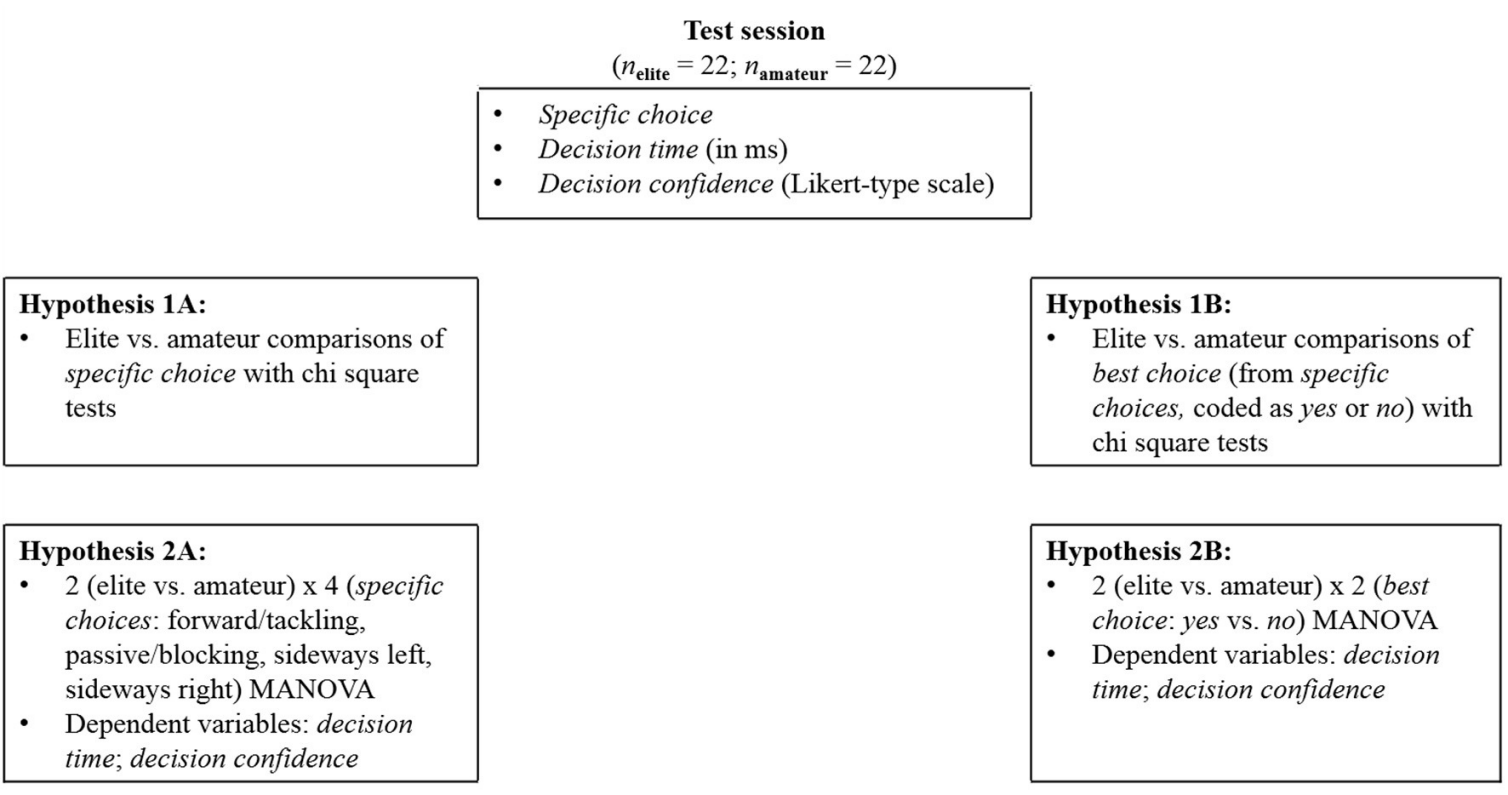
Illustration of the study design including the respective hypotheses.

In particular, we investigated expertise effects in decision-making with a sports-specific embodied-choice paradigm (Lepora and Pezzulo, 2015; Kalén et al., in press). We look at (1) what choices elite players make and (2) how often those choices are the best choices (cf., decision rule of the take-the-best heuristic). By analyzing expertise effects, we also look at the interaction between expertise and the type of choices. By doing so, we can further scrutinize the decision-making processes (i.e., decision time, decision confidence), underlying the elite and amateur players' choices. In detail, we analyze decision time and confidence, because these are theoretically linked and practically crucial for sports choices. To do so, we analyze in a first step which choices elite players made in comparison to amateur players [1a], to then examine how long players of different expertise took to make specific choices, and how confident they were about those [2a]. In a second step, we focus on the best choice in particular [best choice]. In detail, we analyze how often elite players and amateur players made the best choice [1b] as well as how long it took players of different expertise to do so and how confident they were regarding these choices [2b]. Figure 1 provides a brief overview of the created test design.

In detail, we assumed that the elite players would make different embodied choices than amateur players (van Maarseveen et al., 2018b; Magnaguagno and Hossner, 2020) which should be reflected in the frequencies of the *specific choices* [1a]. For *decision time* and *decision confidence*, we expected that, based on their expertise, the elite players would make their embodied choices faster (Raab and Laborde, 2011; Travassos et al., 2013) and with higher confidence (Hepler and Feltz, 2012) [2a].

Further, we predicted higher frequencies of the *best choice* for elite players [1b] based on the team handball study of Magnaguagno and Hossner (2020). According to simple heuristics in sports (Raab, 2012), we could assume that the *best choices* of the elite players are made faster, and elite players are more confident in their selections of the *best choices* than amateurs [2b]. However, there is no empirical evidence so far that compared decision confidence between expertise levels.

## Materials and Methods

### Participants

The participants in this study were part of a previous decision-making experiment (Hinz et al., 2022) in which attack sequences were presented with the temporal-occlusion paradigm instead, and with differing test instructions.

Based on the within–between interaction effects of two previous studies assessing decision-making in realistic setups (Raab and Johnson, 2007; Raab and Laborde, 2011; Bruce et al., 2012), we conducted an *a priori* power analysis for planned multivariate analyses of variances (MANOVA) with global factors (with 1—beta error probability =.95; alpha error probability =.05; the number of groups = 2; response variables = 4; effect size *f* = 0.48 for being the lowest in Raab and Laborde, 2011) using G^*^Power 3.1.9.7 (Faul et al., 2007), revealing a minimum total sample size of 32 participants. Therefore, our total sample size of 44, and the sub-sample sizes of 22 in each group seemed sufficient.

The total sample consisted of 44 male team handball players (*M*_age_ = 19.11 yrs.; *SD* = 6.56 yrs.), who came from four different teams with different performance levels. A total of twenty-two players (*M*_age_ = 17.59 yrs., *SD* = 3.67) were part of a professional youth academy of a First League team handball club competing in the highest possible league within their age category. Based on the definition of Swann et al. (2015), these players can be considered as elite team handball players. A total of twenty-two players (*M*_age_ = 20.71 yrs., *SD* = 8.54) were part of local team handball clubs, competing in non-professional, regional leagues. These players are considered amateur players (Room, 2010). Differences in age between both groups were not significant (*p* = 0.952).

The study received approval by the ethics committee from the local university and met all requirements of the Declaration of Helsinki and its later amendments.

### Apparatus and Stimuli

The experimental setup of the decision-making test was adopted from earlier examinations (for a detailed display of the setup, see Hinz et al., 2021) and applied in an embodied-choice paradigm in a near-game environment. The test scenario, which was presented on a life-size projection screen in front of a contact plate system (SpeedCourt Q12 PRO mobile, GlobalSpeed, Hemsbach, Germany) consisted of attack sequences, showing a center-back player from the view of a central defender. The scenario included video trials with four representative attack actions, and four dummy trial videos (both right-handed attackers) showing too ambiguous actions for an appropriate defense response (for the avoidance of expectation effects; Anderson, 1983). The video clips were all doubled, mirrored (to create a left-hander version of each video trial), and presented in a quasi-randomized order within two blocks, beginning with a right-hander attack block, followed by the left-hander video block, with a 2-min break in-between.

The specific response choices (*forward/tackling; sideways left, sideways right; blocking/passive*) that were prespecified to the contact plates of the SpeedCourt® system were adapted from the previous decision-making test (Hinz et al., 2021). Please refer to this study for detailed explanations regarding response choice mapping and test environment.

The videos were sized 1,280 × 720 pixels (width × height). The test scenario was implemented by using the *Lazarus* (Version 2.0.10) software. In total, the final test scenario consisted of thirty-two video clips of attack sequences of 2 s each, which were presented to the participants during the measurement procedures.

The decision-making test was checked for cross-sectional and longitudinal reliability dimensions (Hinz et al., 2021), which revealed a moderate level of reproducibility. In further statistical analyses, we only used the first presented videos of the attacks (4 right-hander and 4 left-hander attacks) to obtain unbiased responses behavior without memory effects in the participants.

To assess the quality of decisions, we recruited four international team handball experts for a rating of the best tactical choice to finally determine the *best-choice* variable. The four expert raters were characterized by at least 10 years of continuous championship seasons in the German First League, competing for their adult national teams, and achievements in national and international club level titles (European Handball League, European Handball Champions League, European Club Championship, German Championship) as former players and coaches. The rating procedure with the final setup took place before the main experiments. In individual sessions, all four right-hander attack sequences were presented to the experts on the projection screen, and the experts were required to execute a sport-specific motor response on the attacks with one of the four prespecified defense responses. Then, the experts were asked to evaluate the given options for each scene, based on a six-point Likert-type scale (1 = absolutely ambiguous, 2 = ambiguous, 3 = indecisive, 4 = tendentious, 5 = unambiguous, 6 = absolutely unambiguous). In detail, they were asked about the judgment of the best tactical choice and competition-similarity of the shown attacks.

To determine the *best choice*, a “majority rule” procedure according to Johnson and Raab (2003) was applied: For each of the eight video trials, when 3 out of 4 experts evaluated the same option as being the best tactical choice, this decision was coded as the best choice. Given the four baseline stimuli, namely the 4 right-hander attack actions, the experts agreed on the best decision in 75% of the cases. This majority rule is also statistically supported by a chi-square test with Monte Carlo simulation (2,000 replicates), where we found that an observed agreement of three out of four raters differs significantly from chance probability (i.e., 1/4, χ^2^ = 5.33, *p* = 0.049). The raters' selection demonstrated also satisfying judgments for the perceived competition-similarity (*M* = 5.6; *SD* = 0.6). The *best choice* was coded in the database with the *best choice* selected (1 = *yes*) or not (0 = *no*).

### Procedure

Before the start of the single test sessions, written informed consent of the parents of the underage participants was obtained. After an autonomous, game-specific warm-up (about 5 min), explanations about the experimental setting and test procedure were provided by the test staff. Starting with standardized oral instructions, the players were instructed to put themselves in the position of the central block defender in a classic man-to-man defense without teammates, or other opponents than the attacker in the video/situation. Then, four familiarization videos were provided to get used to the test environment. In the main test session, the participants were challenged to execute a direct defense-specific motor response to the attack sequences in the video clips as if they would defend the attacker in a real game situation. Following their first intuition, they were allowed to initialize their motor response whenever required. When a participant left the starting contact plate (central plate), the projection screen turned black for the avoidance of a response bias. After each motor response, players had to give a verbal statement about their self-perceived decision confidence regarding the appropriateness of their made response choice with a six-point Likert-type scale (1 = absolutely ambiguous, 2 = ambiguous, 3 = indecisive, 4 = tendentious, 5 = unambiguous, 6 = absolutely unambiguous). One test session took about 15 min in total.

### Statistical Analysis

Dependent variables were decision time (interval-scaled, in ms), measured as the elapsed time from the start of a video trial till leaving the starting contact plate and decision confidence (interval-scaled, Likert-type scale). In preparation for the analysis, all data points from the eight video trials of the decision-making test were extracted. Based on the total sample size of *N* = 44, we received 352 data points for each variable. Potential outliers were identified based on the absolute deviation around the sample median (MAD, see Leys et al., 2013, for details) of decision time (separately for each action). We defined a moderately conservative rejection criterion of 2.5 times the MAD below or above the median (Leys et al., 2013) to identify decision time outliers in the sample. If a case exceeded the rejection criterion value, all associated variables (i.e., response, decision time, decision confidence, best choice) were discarded from further statistical analyses.

In a first step, we compared which embodied choices were made by elite vs. amateur players with chi-square tests. Specifically, we were interested in the frequencies of the *specific choices* made [1a], and in the frequencies of making the *best choice* [1b]. To aid the interpretation of chi-square test results, we calculated the effect size of Cramer's *V* (Kim, 2017). To this end, the chi-square value was divided by the sample size *n* and then the square root was taken, yielding a value ranging from −1 to 1. We tested the general difference in the frequencies of all four *specific choices* and *best choice* (*yes* vs. *no*) between elite and amateur players as well as single between-group differences in each of the *specific choices*. Cramer's *V* values for the four *specific choices* (*df* = 3) can be interpreted as laid down by Kim (2017): 0.06 < |ϕ| <0.17 “small”, 0.17 < |ϕ| <0.29 “medium”, and |ϕ| > 0.29 “large” effect. Cramer's *V* values for *best choice* (*yes* vs. *no*; *df* = 1) can be interpreted with 0.10 < |ϕ| <0.30 “small”, 0.30 < |ϕ| <0.50 “medium”, and |ϕ| > 0.50 “large” effect (Kim, 2017).

In a second step, we looked at the interaction effects of the different expertise groups and their *specific choices* made [2a], as well as at the interaction effects of the different expertise groups and their *best choices* made [2b] with the decision-making process variables' decision time and decision confidence. This was analyzed with two separate mixed MANOVAs, i.e., a 2 (expertise group_between_: elite vs. amateur) x 4 (specific choice_within_: forward/tackling; passive/blocking; sideways left; sideways right) and a 2 (expertise group_between_: elite vs. amateur) × 2 (best choice_within_: yes vs. no) with the dependent variables decision time and decision confidence. If significant multivariate main and interaction effects were obtained, those were followed up with subsequent univariate analyses to check which of the obtained dependent variables were affected. The assumption checks revealed that the Box's M-test for homogeneity of covariances was not significant for the *specific choice* MANOVA, but significant in the *best choice* MANOVA (*p* = 0.009). Shapiro-Wilk's tests for multivariate normality were violated (both *p*s < 0.001), however, we conducted both MANOVAs in accordance with the guidelines of Finch (2016). Cohen's *d* was calculated as a measure of effect size with interpretations against the following scale: 0.2 > *d*, trivial; 0.2 ≤ *d* < 0.5, small; 0.5 ≤ *d* < 0.8, moderate; 0.8 < *d*, large (Cohen, 1988). The significance level was set at α = 0.05. Data analyses were conducted using Statistical Package for the Social Sciences Version 26 (SPSS Inc., Chicago, IL, United States).

## Results

### Specific Choices

**Figure 2:**
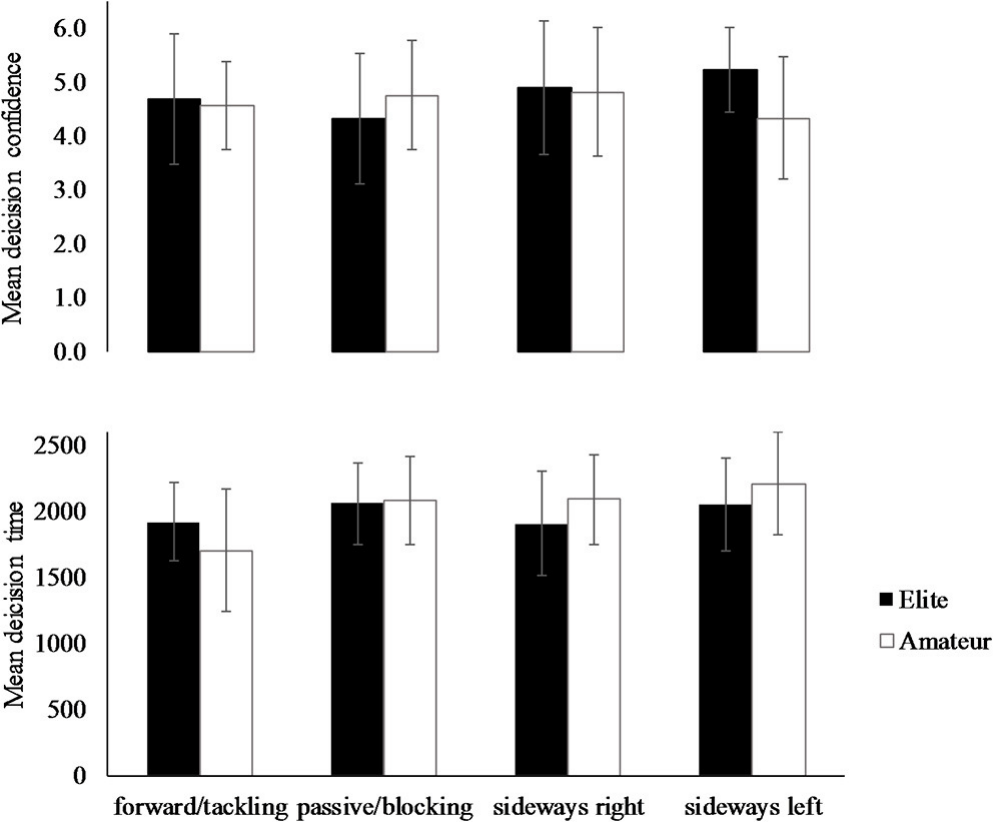
Decision time (in ms) and decision confidence (Likert-type scale) of specific choices of elite and amateur players. Error bars indicate standard deviation.

Descriptive statistics of the *specific choices* and related decision times and decision confidences are presented in Table 1; Figure 2.

**Table 1:** Frequency of specific choice and the respective decision time (in ms) and decision confidence (Likert-type scale) in both player groups.

**Group**	**Specific choice**	**Frequency (*n*; %)**	**Decision time (*M* ±*SD*)**	**Decision confidence (*M ±SD*)**
**Elite**				
	Forward/tackling	72 (44.7%)	1,920 ± 298	4.7 ± 2.0
	Passive/blocking	69 (42.9%)	2,058 ± 305	4.3 ± 1.2
	Sideways right	11 (6.8%)	1,909 ± 393	4.9 ± 1.2
	Sideways left	9 (5.6%)	2,049 ± 349	5.2 ± 0.8
**Amateur**				
	Forward/tackling	32 (20.6%)	1,704 ± 459	4.6 ± 0.8
	Passive/blocking	96 (62.6%)	2,084 ± 332	4.8 ± 1.0
	Sideways right	11 (7.1%)	2,092 ± 340	4.8 ± 1.2
	Sideways left	15 (9.7%)	2,211 ± 387	4.3 ± 1.1
**Both**				
	Forward/tackling	104 (33.3%)	1,853 ± 370	4.6 ± 1.1
	Passive/blocking	165 (6.7%)	2,073 ± 356	4.6 ± 1.1
	Sideways right	22 (52.4%)	2,000 ± 387	4.9 ± 1.1
	Sideways left	24 (7.6%)	2,150 ± 389	4.7 ± 1.3

#### Frequencies

The statistical evaluation showed that elite players used forward/tackling most frequently (44.7%), and sideways left least frequently (5.6%). Amateur players used passive/blocking most frequently (62.9%) and sideways right least frequently (7.1%).

Testing for expertise effects in specific choice (Hypothesis 1a), significant chi-square test results showed that elite and amateur players indeed differed in how often they made *specific choices*, χ^2^_(3)_ = 21.501, *p* < 0.001, Cramer's *V* = 0.26. In particular, single between-group differences in forward/tackling [$\chi_{\left(1\right)}^{2}$ = 8.94, *p* = 0.004] and passive/blocking [$\chi_{\left(1\right)}^{2}$ = 21.84, *p* < 0.001] were also revealed, while other choice frequencies (i.e., sideways right and left, *p*s > 0.05) did not differ significantly between expertise groups.

The MANOVA analysis of whether the decision-making processes of elite and amateur players differed based on their *specific choices* made (i.e., expertise × specific choice interaction; Hypothesis 2a) did not show the main effect of *expertise* [Wilks' λ = 1.00, *F*_(1, 308)_ = 0.46, *p* = 0.663] but revealed a significant main effects of *specific choice* [Wilks' λ = 0.91, *F*_(1, 308)_ = 4.89, *p* < 0.001] and a significant *expertise* × *specific choice* interaction [Wilks' λ = 0.94, *F*_(3, 308)_ = 3.24, *p* = 0.004].

#### Decision Time and Confidence

Following up the significant multivariate main effect for *specific choice* and the *expertise* × *specific choice* interaction, subsequent univariate analyses for *decision time* showed that *specific choice* [*F*_(3, 308)_ = 8.79, *p* < 0.001, $\eta_{p}^{2}$ = 0.106] and the *expertise* × *specific choice* interaction [*F*_(1, 308)_ = 3.25, *p* = 0.022, $\eta_{p}^{2}$ = 0.036] were significant. In detail, scrutinizing the main effect of *specific choice* revealed that the forward/tackling choice was significantly faster than the passive/blocking choice [*t*_(267)_ = −5.140, *p* < 0.001, *d* = 0.65] and the sideways left choice [*t*_(126)_ = −3.504, *p* < 0.001, *d* = 0.80]. All other *specific choice* comparisons were not significant. Following up the significant *expertise x specific choice* interaction revealed only that elite players were significantly slower than amateur players in their forward/tackling choice [*t*_(102)_ = −2.840, *p* = 0.005, *d* = −0.60] while there were no other significant effects of expertise.

Univariate analyses for *decision confidence* revealed only a significant *expertise* x *specific choice* interaction [*F*_(1, 308)_ = 3.08, *p* = 0.028]. Elite players were less confident in their passive/blocking choice than amateur players [*t*_(163)_ = 2.536, *p* = 0.012, *d* = 0.40]. No significance was revealed in the all other *specific choice* comparisons (Table 1; Figure 2).

### Best Choice (i.e., Decision Rule of Take-the-Best Heuristics)

**Figure 3:**
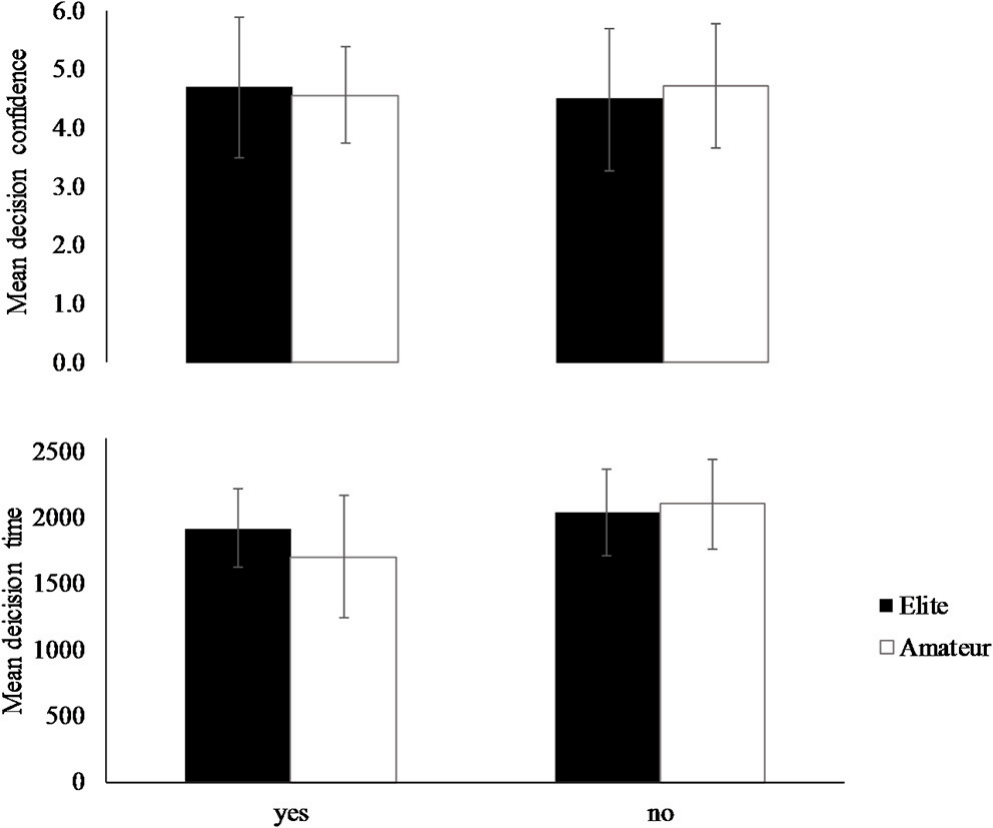
Decision time (in ms) and decision confidence (Likert-type scale) of the selected (yes) and non-selected (no) best choice of elite and amateur players. Error bars indicate standard deviation.

Descriptive statistics of the best choices and related decision times and decision confidences are presented in Table 2; Figure 3.

**Table 2:** Frequencies of selected (yes) and non-selected (no) best choice, and respective decision times (in ms) and confidences (Likert-type scale) in amateur and elite players.

**Group**	**Best choice frequency** **(*****n*****, %)**		**Decision time** **(*****M*** **±*****SD*****)**		**Decision confidence** **(*****M*** **±*****SD*****)**	
	**Yes**	**No**	**Yes**	**No**	**Yes**	**No**
Elite	72 (44.7%)	89 (56.3%)	1,920 ± 298	2,039 ± 325	4.7 ± 1.2	4.5 ± 1.2
Amateur	32 (20.8%)	121 (79.2%)	1,704 ± 459	2,099 ± 315	4.6 ± 0.8	4.8 ± 1.0
Both	104 (33.1%)	210 (66.9%)	1,979 ± 369	2,074 ± 337	4.7 ± 1.1	4.6 ± 1.1

#### Frequencies

For the analyses of the *best choice* (i.e., take-the-best prediction) in the sport-specific embodied-choice paradigm, we checked the frequency of the best options chosen in both expertise groups (Hypothesis 1b). The respective chi-square tests revealed that indeed elite players (44.7 %, *n* = 72) made the *best choice* more often than amateur players (20.8 %, *n* = 32), $\chi_{\left(1\right)}^{2}$ = 20.400, *p* < 0.001, Cramer's V = 0.25.

The MANOVA analysis of whether the decision-making processes of elite and amateur players differed based on the *best choices* made (i.e., expertise × best choice interaction; Hypothesis 2b) did not show a main effect of *expertise* [Wilks's λ = 1.00, *F*_(1, 311)_ = 0.62, *p* = 0.538], but revealed significant main effects of *best choice* [Wilks's λ = 0.91, *F*_(1, 311)_ = 15.19, *p* < 0.001] and the *expertise* × *best choice* interaction [Wilks's λ = 0.97, *F*_(1, 311)_ = 5.27, *p* = 0.006].

#### Decision Time and Confidence

Following up the significant multivariate main effect and interaction, the subsequent univariate analyses for *decision time* showed significance in *best choice* [*F*_(1, 311)_ = 28.32, *p* < 0.001, $\eta_{p}^{2}$ = 0.101] and the *expertise* × *best choice* interaction [*F*_(1, 311)_ = 10.14, *p* = 0.002, $\eta_{p}^{2}$ = 0.032]. In detail, *t*-tests revealed significant faster decision times of when the best choice was selected compared to when it was not selected [*t*_(313)_ = 5.290, *p* < 0.001, *d* = 0.63], and elite players showed slower *decision times* than amateurs when they selected the *best choice* [*t*_(102)_ = −2.840, *p* = 0.005, *d* = −0.60]. No between-group difference was revealed in the non-selected *best choice* [*t*_(209)_ = 1.324, *p* = 0.187, *d* = 0.18].

Univariate analyses for *decision confidence* revealed no significant affections of *expertise* [*F*_(1, 311)_ = 0.76, *p* = 0.384, $\eta_{p}^{2}$ < 0.001], *best choice* [*F*_(1, 311)_ = 0.20, *p* = 0.656, $\eta_{p}^{2}$ < 0.001], and the *expertise* x *best choice* interaction [*F*_(1, 311)_ = 1.49, *p* = 0.224, $\eta_{p}^{2}$ = 0.005] (Table 2; Figure 3).

## Discussion

With respect to the methodological issues in decision-making and simple heuristic experiments, and current concepts in this very field, we carried out a sport-specific embodied-choice experiment with elite and amateur team handball players. Thereby, we extend recent knowledge of expertise effects in decision-making from an embodied-choice perspective (Lepora and Pezzulo, 2015; Raab, 2017). Within this study, we looked at the *specific* and *best choices* players made, and we also scrutinized the decision-making processes (i.e., decision time, decision confidence) underlying the elites' and amateurs' choices. Against previous assumptions in simple heuristic and decision-making literature (Williams et al., 2004; Raab and Laborde, 2011), we revealed an expertise-related speed-accuracy tradeoff showing that elite players seem to sacrifice decision time for higher decision quality.

### Specific and Best Embodied Choices Are Expertise-Related

In line with our assumptions based on previous decision-making experiments with sport-specific motor responses (van Maarseveen et al., 2018b; Magnaguagno and Hossner, 2020), we found an overall difference between expertise groups in the frequencies of the *specific choices*, showing highest frequencies for forward/tackling choices for the elite players, whereat amateurs chose passive/blocking most frequently.

Closer examinations of the differing frequencies of *specific choices* revealed a discrepancy in the choices of forward/tackling and passive/blocking between elite and amateur players, supporting expertise-specific decision-making in sports (Travassos et al., 2013; Araújo et al., 2019). While sideways left and sideways right choices played a less important role for both groups alike. Elite players chose forward/tackling more frequently (Δ 24.1%) than amateurs, who chose passive/blocking more frequently (Δ 19.7%). This implies a level-depending judgment of the need to intervene with an active defense action (i.e., forward/tackling) as opposed to a passive one (i.e., passive/blocking). One reason for that can be found in the differing decision confidences between elite and amateur players in the passive/blocking choice. Elite players are significantly less confident (4.3 ± 1.2) than amateurs (4.8 ± 1.0) in this particular choice indicating that more confidence seems to go along with less risk taking of amateurs, culminating in varying decisions between groups. Elite players seem to have perceived passive/blocking as riskier than a forward/tackling choice, because staying passive or blocking might allow the attacker to approach closer toward the goal, and duels in their defense, the near-goal area can be riskier. Therefore, elite players chose the rather active option of forward/tackling to potentially prevent increasing pressure from the opponent attacker toward the goal. Our hypothesis of higher confidence in elite players was not confirmed, but the fact that specific choices and decision confidence are linked confirms the connection between decision-making performance confidence and first option as demonstrated by Hepler and Feltz (2012) in basketball.

Furthermore, we hypothesized based on simple heuristics and embodied-choice research (Raab and Laborde, 2011; Raab, 2012; van Maarseveen et al., 2018b; Magnaguagno and Hossner, 2020) to find higher quality of choices in elite players. This was confirmed by significantly more frequent *best choices* of elite players in comparison to amateur players (Δ 24.9%). In line with our findings, a previous team handball study did also find higher correctness of responses by expert players (Magnaguagno and Hossner, 2020). The results for the *best* (and *specific) choice* lend support to the general tenets of embodied choices (Lepora and Pezzulo, 2015).

One reason for elite players making different and also better choices than amateurs might be due to their knowledge and experience with defensive tactics. The test instructions provided explanations about a man-to-man defense system players had to see themselves put in. Certain rules within this defense system apply, which are taught in basic practice lessons from early team handball ages on (Pabst and Scherbaum, 2018). With increasing age and expertise level, players not only practice more often and compete higher, but they also learn and adapt defense systems further. Enhanced situation-specific learning effects foster the knowledge of response consequences (Raab, 2014), which can enable elite players to decide better in tactical situations. From an ecological perspective, elite players could also rely more on their physical ability to move fast during play, which would allow them to invest more time for an advantageous duration extension of the information pick up.

Broadly speaking, our created performer environment setting that involves sport-specific motor responses was able to unfold expert decision-making differences within a sport-specific embodied-choice test. Since cognitive and motor components of choices seem to be intertwined (Raab, 2017), the sport-specific motor responses in our study include the interactions of both of these components. This appears to reflect an athlete's actual decision-making performance better than uncoupled perception-action experiments due to the consideration of a representative task design for the experimental representations (Brunswik, 1956; Araújo et al., 2007; Dicks et al., 2009; Travassos et al., 2013).

### Embodied Choices of Elite Players Are Slower and Better

To increase the ecological validity of experimental findings in decision-making, our sport-specific embodied-choice test offers possibilities to evaluate *what* (choices) and *how* (decision time) to act (Raab, 2017). Here, we analyzed the expertise differences in *specific* and *best choices* and how these choices were made. To do so, we analyzed the decision time and confidence.

The general range of decision time data is similar to those from the *in situ* experiment in basketball (van Maarseveen et al., 2018b), where the time of correct and incorrect decisions show equal proportions to the time data of our obtained *best choices*. This delivers support for the representative task design of our experimental approach.

Most notably in our study, we revealed slower decision times of *specific choices* (i.e., forward/tackling) and *best choice* of elite players, which stands in contrast to our assumptions of faster and more accurate decisions of better athletes from simple heuristics and decision-making research (Raab and Laborde, 2011; Raab, 2012; Travassos et al., 2013). The slower decision times of the elite players are possibly a result of *corrective* top–down processes interacting with bottom-up processes (Raab, 2014) of the sensorimotor system during decision-making. *Corrective* interactions are action preparations of an athlete toward an opponent's action preference (top–down), however, with the ongoing course of the event, the final choice depends more on additional cues perceived in the unfolding action of the opponent (bottom-up). We assume that the players' embodied choices underly level-dependent *corrective* interactions during the decision-making processes, leading to slower, but therefore, higher quality decisions of elite players. Corrective interactions, as a dynamic function, may also rely on previous successful experiences (Raab, 2014).

Transferring this theory to our test setting, we believe that the perceived kinematic cues of the approaching attackers in the videos, such as run-up speed and proximity to the defender, first provoked an intuitive action preparation in our players (Raab and Laborde, 2011). With the ongoing time-motion course, the players perceive further kinematic cues of the attackers, such as preparing throwing kinematics or head movements, which seem to be judged differently by elite players and amateur players. Elite players could invest additional time within the top–down bottom-up control to “wait-and-see” what choice would be the best in this particular moment. This means that the quality of choices is prioritized above the speed. These fine-grained expertise differences in the advantageous usage of kinematic information were also proven in striking sports, as discussed in the review of Morris-Binelli and Müller (2017).

A general phenomenon in sports that underpins such time investments in favor of higher quality is the so-called *speed-accuracy tradeoff* (Schmidt and Lee, 2005), meaning that a more time-consuming evaluation of a situation can lead to higher success rates and fewer errors (Johnson, 2006). The slower but more frequent *best choices* by the elite players indicate an expertise-related effect of speed-accuracy tradeoffs (Ratcliff et al., 2016) between both groups. Due to the elite players' extensive knowledge from increased training amount, higher competition levels, and (successful) experiences (Raab, 2014), this information seems to be processed during the top–down and bottom-up interactions requiring longer processing times. Investigations on speed-accuracy tradeoffs in multialternative decision-making setups are rare. One investigation in rugby showed similar effects with a full-body interception or “tackle” responses (Brault et al., 2012). In boxing, Ottoboni et al. (2015) showed a similar tradeoff in experts for reaction time and punching hand identification in a computer-based experiment when compared to beginners or novices. From an anticipation perspective, the speed-accuracy tradeoff in the elite players could also be related to more pronounced task-specific expertise within their action–observation network (system of brain structures with “mirroring” abilities) (Balser et al., 2014). Stronger neural activation within several sections of the action–observation network for superior action anticipation performance could be forwarded to the sensorimotor interactions during decision-making processes, ultimately leading to superior decision quality (as a function of decision accuracy and time) in the elite players.

In a nutshell, the findings for decision time do not fully support previous research on simple heuristics. In fact, contrary to faster and better tactical choices of better players (Williams et al., 2004; Raab and Laborde, 2011; Travassos et al., 2013), we illustrate the reverse relation between the *best choice* and *decision time*. Supposedly, decision-making performance depends on factors such as decision time and confidence, meaning that better athletes make better decisions but in a slower way. We assume decision time and confidence to play a more crucial and differential role in making embodied choices underlining the necessity for further experiments within this framework.

### Limitations

Even though the sport-specific embodied-choice test was able to scrutinize complex sports behavior through its applied environment and the sport-specific motor components involved, it remains open if equal performances would have been achieved in a non-motor test environment as well. As literature presents, an embodied choice must not automatically overt motor responses (Aglioti et al., 2008). In the same vein, a comparable *in situ*, on-field performance test, as carried out by van Maarseveen et al. (2018a) in soccer would give more clarifications about the ecological validity of the obtained test results.

Furthermore, this study did not consider isolated cognitive skills and gaze behavior of the players, which would have provided additional information about information pick up and processing strategies. As presented in the literature, experts in sports possess superior abilities in executive control, visuospatial tasks, and perception of movement patterns (Abernethy and Zawi, 2007; Alves et al., 2013; Ottoboni et al., 2021) that could also impact complex sport behavior.

To further increase the validity of the paradigm, additional choices for the players would represent the individual choice of each player better. While the responses in the test are determined as linear movements, defending in-game situations is always a unique event, since kinematic information constantly affects anticipation and decision-making (Gredin et al., 2020). For this reason, movements are not always linear, they rather follow a non-linear trajectory. Some players, therefore, showed first upper body sways in preparation for a forward/tackling choice in their starting position, but by perceiving a “mind-changing” cue in the attacker's ongoing action, the player changed his mind to decide on a sideways movement instead. Such a short-term change within the players' movement was not measured, but it indicated the non-linearity of the motor execution of an embodied choice.

### Implications

This study adds new insights to the understanding of embodied choices in sports, especially with respect to decision time and confidence.

For theory, our results imply that decision-making research should henceforth consider not only *what* decisions are made, but also *how* these decisions are made in the same vein. Based on our results and general decision-making theories, decision time and decision confidence can be considered to better understand the decision-making processes in sports and beyond. This might help in specifying the exact mechanisms and existing theories applied in the sports context that do consider the decision-making process itself (not only the outcome), such as simple heuristics.

In particular, regarding decision time in our study revealed that elite players made better but slower embodied choices. In the sense of a speed-accuracy tradeoff, they seemed to prioritize the quality. This finding has important applied implications: If speed accuracy plays a role in sport-specific embodied choices, players and coaches should focus more on the accuracy of a choice in defense decisions.

For the applied field, we recommend that close-to “real-world” experimental setups such as the sport-specific embodied-choice test presented here could be applied as a tactical training tool in team handball and other team sports. Similarly, a row of longitudinal 3D-video-based interventions to train team handball tactics were conducted in the past (Raab et al., 2008). Results demonstrate improvements in tactical decision-making of players, facilitated by 3D-presentations of attack situations. We hope that the sport-specific embodied-choice test presented here which has been shown to produce reliable outcomes (Hinz et al., 2021) will be used similarly to improve tactical decision-making training in sports and the embodied choices of players in the future.

## Conclusion

In conclusion, this study demonstrated the importance of capturing motor responses and the considerations of decision time and decision confidence within the embodied-choice experiments. The sport-specific embodied-choice test presented here revealed differences in choices between elite and amateur team handball players, confirming recent team sports studies with embodied choices (van Maarseveen et al., 2018b; Magnaguagno and Hossner, 2020), and indicating expertise-related decision-making (Raab, 2012; Travassos et al., 2013; Magnaguagno and Hossner, 2020). However, slower decision times for *specific* and *best choices* were found, which is in contrast to experts' faster and better choices in other sports studies (Williams et al., 2004; Raab and Laborde, 2011; Travassos et al., 2013). We, therefore, provide possible explanations why better athletes made better choices slower, instead of faster: Corrective top–down bottom-up interactions (Raab, 2014), and speed-accuracy tradeoffs (Ratcliff et al., 2016) in favor of accuracy could be the reasons for additional time investments by elite players to select a better-embodied choice. In general, fairly high decision confidence in both groups emphasizes subjective accuracy of their decisions, but between-group confidence differences in particular choices (passive/blocking) point to the impact of confidence on decision-making performance. Together, this study provides further empirical evidence bearing up on the embodied-choice framework in sports and, especially, highlights the importance of better understanding the mechanism underlying embodied choices, such as through decision time and confidence.

## Data Availability Statement

The raw data supporting the conclusions of this article will be made available by the authors, without undue reservation.

## Ethics Statement

The studies involving human participants were reviewed and approved by Local Ethics Committee from the Otto von Guericke University Magdeburg/Germany. Written informed consent to participate in this study was provided by the participants' legal guardian/next of kin.

## Author Contributions

MH, NL, and LM: conceptualization, software, data curation, and formal analysis. NL: funding acquisition and validation. MH: investigation, resources, supervision, and writing—original draft. MH and LM: methodology, project administration, and visualization. NL and LM: writing—review and editing. All authors contributed to the article and approved the submitted version.

## Funding

This work was supported by the Federal Institute of Sport Science (IIA1- 070506/19-20). The funders had no role in the study design, data collection and analysis, and decision to publish or preparation of the manuscript.

## Conflict of Interest

The authors declare that the research was conducted in the absence of any commercial or financial relationships that could be construed as a potential conflict of interest.

## Publisher's Note

All claims expressed in this article are solely those of the authors and do not necessarily represent those of their affiliated organizations, or those of the publisher, the editors and the reviewers. Any product that may be evaluated in this article, or claim that may be made by its manufacturer, is not guaranteed or endorsed by the publisher.
